# *Acanthamoeba* and *Dictyostelium* as Cellular Models for *Legionella* Infection

**DOI:** 10.3389/fcimb.2018.00061

**Published:** 2018-03-02

**Authors:** A. Leoni Swart, Christopher F. Harrison, Ludwig Eichinger, Michael Steinert, Hubert Hilbi

**Affiliations:** ^1^Institute of Medical Microbiology, Medical Faculty, University of Zurich, Zurich, Switzerland; ^2^Max von Pettenkofer Institute, Medical Faculty, Ludwig-Maximilians University Munich, Munich, Germany; ^3^Institute for Biochemistry I, Medical Faculty, University Hospital Cologne, Cologne, Germany; ^4^Department of Life Sciences, Institute of Microbiology, Technical University of Braunschweig, Braunschweig, Germany

**Keywords:** amoebae, effector protein, GTPase, host-pathogen interaction, pathogen vacuole, phosphoinositide lipid, retrograde transport, type IV secretion

## Abstract

Environmental bacteria of the genus *Legionella* naturally parasitize free-living amoebae. Upon inhalation of bacteria-laden aerosols, the opportunistic pathogens grow intracellularly in alveolar macrophages and can cause a life-threatening pneumonia termed Legionnaires' disease. Intracellular replication in amoebae and macrophages takes place in a unique membrane-bound compartment, the *Legionella*-containing vacuole (LCV). LCV formation requires the bacterial Icm/Dot type IV secretion system, which translocates literally hundreds of “effector” proteins into host cells, where they modulate crucial cellular processes for the pathogen's benefit. The mechanism of LCV formation appears to be evolutionarily conserved, and therefore, amoebae are not only ecologically significant niches for *Legionella* spp., but also useful cellular models for eukaryotic phagocytes. In particular, *Acanthamoeba castellanii* and *Dictyostelium discoideum* emerged over the last years as versatile and powerful models. Using genetic, biochemical and cell biological approaches, molecular interactions between amoebae and *Legionella pneumophila* have recently been investigated in detail with a focus on the role of phosphoinositide lipids, small and large GTPases, autophagy components and the retromer complex, as well as on bacterial effectors targeting these host factors.

## *Legionella* spp.—environmental bacteria and opportunistic pathogens

**Figure 1:**
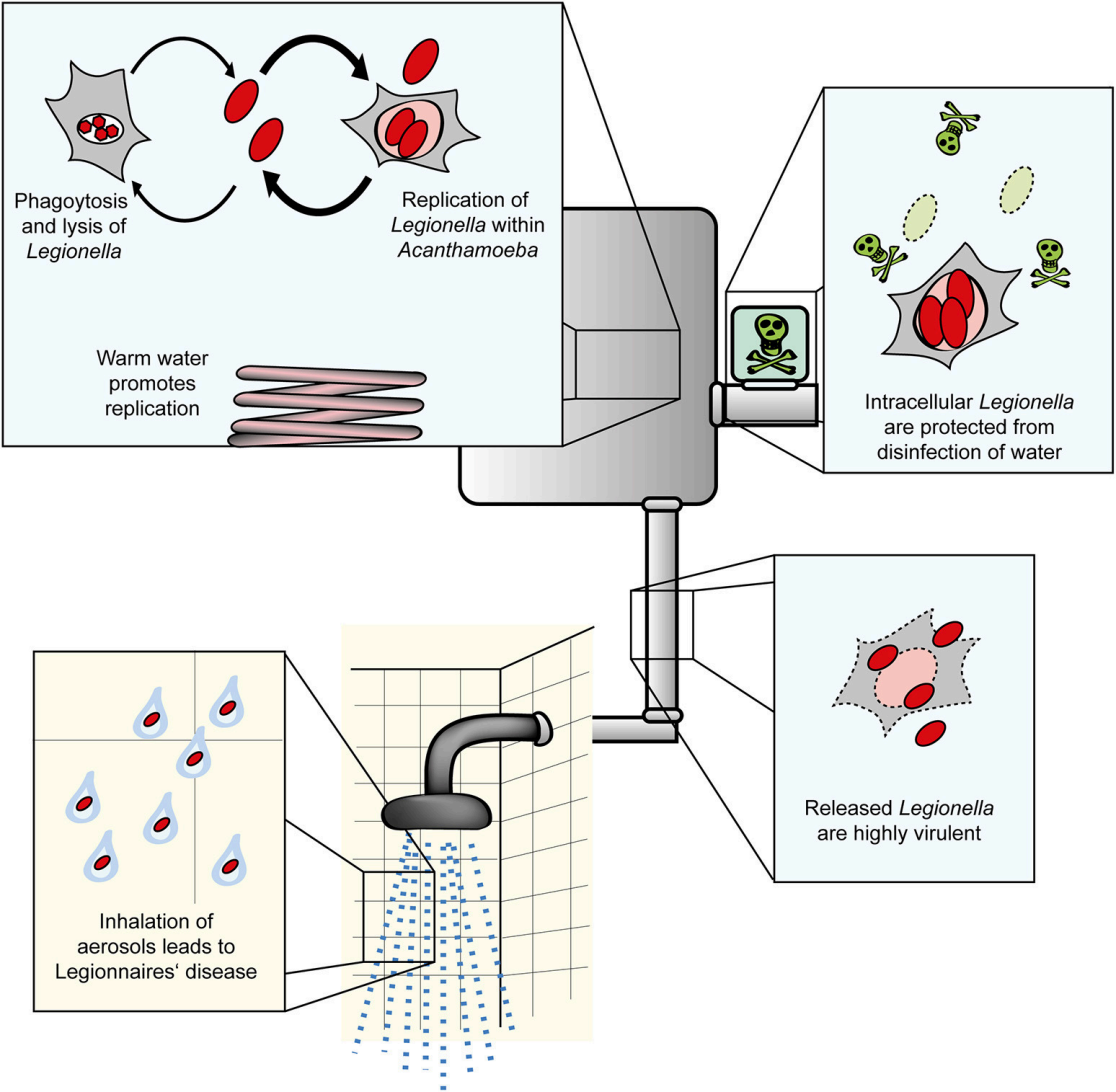
Amoebae as natural reservoir for *Legionella* in water systems. *L. pneumophila* and amoebae are commonly isolated from natural and technical water systems. At cooler temperatures (below ca. 20–25°C), *L. pneumophila* resists *A. castellanii*, but does not or only slowly replicate in the amoebae. At elevated temperatures occurring in technical water systems, *L. pneumophila* efficiently replicates in the amoebae. Internalized *Legionella* bacteria are less susceptible to routine disinfection and released from the amoebae in a highly virulent form. Virulent bacteria present in aerosols are inhaled, infect alveolar macrophages and cause Legionnaires' disease.

*Legionella* spp. are the causative agents of a potentially fatal pneumonia termed Legionnaires' disease. Overall, *Legionella pneumophila* is the clinically most relevant species, followed by *Legionella longbeachae*, which is associated with outbreaks of Legionnaires' disease particularly in Australia and New Zealand (Newton et al., 2010). The Gram-negative bacteria are ubiquitously found in the environment, where they inhabit natural and man-made freshwater systems. Upon inhalation of *Legionella*-laden aerosols, the opportunistic pathogenic bacteria enter the human lung, where they infect alveolar macrophages and might cause a fulminant pneumonia (Figure 1). *Legionella* spp. are usually regarded to only accidentally infect humans after transmission by technical vectors such as cooling towers, fountains, or showers (Benin et al., 2002; Newton et al., 2010; Hilbi et al., 2011; Yamaguchi et al., 2017). However, recently the first probable transmission of a highly virulent *L. pneumophila* strain from person-to-person was reported (Correia et al., 2016).

*L. pneumophila* replicates intracellularly within a unique membrane compartment, which is called the *Legionella*-containing vacuole (LCV). This compartment avoids acidification and fusion with lysosomes, but extensively communicates with multiple vesicle trafficking pathways, including the endosomal, secretory and retrograde route, and after a series of maturation steps associates with the endoplasmic reticulum (ER) (Isberg et al., 2009; Asrat et al., 2014; Finsel and Hilbi, 2015; Personnic et al., 2016; Bärlocher et al., 2017b). LCV formation is mechanistically similar in amoebae and macrophages and requires the Icm/Dot (Intracellular multiplication/Defective organelle transport) type IV secretion system (T4SS) (Segal et al., 1998; Vogel et al., 1998; Segal and Shuman, 1999; Hägele et al., 2000; Solomon et al., 2000). This T4SS of *L. pneumophila* translocates ~300 different “effector” proteins into host cells, wherein these virulence factors subvert pivotal processes such as signal transduction, cytoskeleton dynamics, and membrane trafficking (Isberg et al., 2009; Hubber and Roy, 2010; Hilbi and Haas, 2012; Haneburger and Hilbi, 2013; Sherwood and Roy, 2013; Finsel and Hilbi, 2015; Qiu and Luo, 2017). The Icm/Dot T4SS (but not the “effectorome”) is conserved in the genus *Legionella*, including *L. longbeachae*, where it is also essential for virulence in amoebae and mice (Cazalet et al., 2004, 2010; Chien et al., 2004). Of note, the bioinformatics analysis of the *Legionella* genome sequences revealed a number of genes encoding eukaryotic-like proteins or motifs, which likely subvert host cell functions in order to allow the pathogen to survive and replicate intracellularly (Cazalet et al., 2004, 2010; Chien et al., 2004).

## Protozoa as environmental niches of *Legionella* spp.

Environmental microorganisms commonly colonize and form communities called biofilms, which comprise large numbers of prokaryotic and eukaryotic cells associating via adhesion molecules and secreted compounds. Biofilms are ubiquitously found in the environment and contain a plethora of bacterial species, many of which communicate via low molecular weight signaling molecules in a process termed quorum sensing (Hall-Stoodley et al., 2004; Hochstrasser and Hilbi, 2017). These biofilms are attacked (“grazed”) by predatory amoebae and ciliates, resulting in a reduction in the bacterial population. In contrast to most other bacterial genera, *Legionella* spp. resist degradation by amoebae and other protozoa (Newton et al., 2010; Hilbi et al., 2011).

In its aquatic habitats *L. pneumophila* replicates intracellularly in various free-living protozoa, including amoebae such as *Acanthamoeba, Vermamoeba* (formerly *Hartmannella*), *Naegleria, Echinamoeba*, and *Vahlkampfia*, as well as in ciliates like *Tetrahymena* (Fields, 1996; Greub and Raoult, 2004; Hilbi et al., 2011; Hsu et al., 2015). From an evolutionary point of view, interactions of *L. pneumophila* with protozoa shaped the relationship and likely led to adaptive responses, which enable the pathogen to also infect mammalian phagocytes such as human alveolar macrophages (Greub and Raoult, 2004; Molmeret et al., 2005; Jäger et al., 2014). In addition to their role as natural reservoir and selection niche of virulence traits, protozoa can also enhance the transmission of *L. pneumophila* either as intact host cell or as expelled vesicles filled with bacteria (Rowbotham, 1986; Brieland et al., 1997; Amaro et al., 2015).

A number of studies indicated that *L. pneumophila* is highly adapted and manages to infect a wide range of protozoan and metazoan hosts (Hilbi et al., 2011; Bergmann and Steinert, 2015; Hsu et al., 2015). This suggests that many protozoan host species and other interaction partners of *L. pneumophila* in the environment remain to be discovered. Recently, we reported that *L. pneumophila*, protozoa and aquatic nematodes thrive in close association within biofilms (Rasch et al., 2016). Microscopic inspection of biofilms and inoculation experiments with mCherry-labeled *L. pneumophila* identified the ciliates *Oxytricha bifaria, Stylonychia mytilus*, and *Ciliophrya* sp. as potential new protozoan hosts of *L. pneumophila*. While several metazoan organisms were identified in the biofilm (Copepoda, Nauplius larvae, Rotifera, and nematodes), only the nematodes accumulated *L. pneumophila* within their intestines. This correlates with the finding that the nematode *Caenorhabditis elegans* can be infected with *L. pneumophila* under laboratory conditions (Brassinga et al., 2010; Komura et al., 2010).

To study the *Legionella*-protozoa-nematode interactions, we infected axenically grown *Acanthamoeba castellanii* and *C. elegans* with *L. pneumophila*. This model system indicated that nematode larvae rupture the infected amoebae cells and thus are exposed to *L. pneumophila* (Rasch et al., 2016). The interaction may be different for *Legionella*-filled amoebae cysts and spores. *D. discoideum* spores resist destruction by the pharyngeal grinders of nematodes such as *C. elegans* and seem to be disseminated by the nematode (Kessin et al., 1996). Thus, if infected with *Legionella*, cysts and spores might disseminate the pathogen along this route.

*L. pneumophila* grown in amoebae shows distinct features compared to bacteria grown in broth (Figure 1). Accordingly, upon growth in *Acanthamoeba polyphaga* the bacteria are more tolerant to antibiotics (Barker et al., 1995), while grown in *A. castellanii* the bacteria are more virulent compared to growth on agar plates (Cirillo et al., 1994). Furthermore, the amoeba species within which replication occurs seems to also play a role, such that resistance to chlorination is higher in bacteria released from *Hartmanella vermiformis* than from *A. castellanii* (Chang et al., 2009).

## Evolution and gene regulation of *Legionella* in amoebae

*Legionella* spp. are known to undergo horizontal gene transfer (Miyamoto et al., 2003). Gene transfer between bacteria has been shown to be significantly enhanced within the digestive vacuole of protozoa, presenting the possibility that they may act as a “trading post” to allow the acquisition of virulence genes (Schlimme et al., 1997). Perhaps reflecting this process, *L. pneumophila* contains a number of genes believed to be derived from eukaryotic, bacterial, or even viral sources (Lurie-Weinberger et al., 2010). These findings suggest that amoebae can be considered as an evolutionary niche, wherein the selective pressure and the potential for *Legionella* to acquire new virulence-related genes is higher than in the extracellular environment (Greub and Raoult, 2004; Molmeret et al., 2005).

An analysis of the *L. pneumophila* transcriptome under different growth conditions revealed that expression of almost half the genome is altered upon shifting from replicative to transmissive phase (Brüggemann et al., 2006). This was observed for bacteria grown in either broth cultures or in *A. castellanii*. The transmissive phase, which occurs following completion of intracellular replication, is characterized by upregulation of virulence and invasion genes, including substrates of the Icm/Dot T4SS and motility genes (Brüggemann et al., 2006). The gene regulation of *L. pneumophila* is further complicated by the existence of non-coding (nc) RNAs, which have been shown to play a role in regulation of virulence traits (Romby et al., 2006). *L. pneumophila* contains a number of these regulatory ncRNAs, which likely are implicated in virulence, since their expression changes during the bacterial biphasic life cycle or upon infection of *A. castellanii* (Weissenmayer et al., 2011).

## *Acanthamoeba* as a natural and model host of *Legionella*

The distinct features of the protozoan genera *Acanthamoeba* and *Dictyostelium* have been particularly useful in examining the ecology and cellular host-interactions of *L. pneumophila*. Given the similarity of the infection process in amoebae and macrophages, the amoebae are powerful models to study bacteria-macrophage interactions (Hilbi et al., 2007; Escoll et al., 2013; Hoffmann et al., 2013; Bergmann and Steinert, 2015). *Acanthamoeba* has often been found in *Legionella*-positive habitats and has an extremely wide distribution. The amoebae have been isolated from diverse aquatic environments, including soil (Sawyer, 1989), roadside puddles (Sakamoto et al., 2009), fresh water lakes and rivers (John and Howard, 1995), frozen lakes (Brown and Cursons, 1977), the atmosphere (Rodriguez-Zaragoza et al., 1993), and even the Antarctic (Brown et al., 1982).

The majority of *Acanthamoeba* isolates from these aquatic environments harbor endosymbionts, such as viruses, bacteria, yeast, and protists (Greub and Raoult, 2004). However, for laboratory studies of host-pathogen interactions *Acanthamoeba* strains adapted to axenic growth are mostly used (Shevchuk et al., 2014; Eisenreich and Heuner, 2016). Like many other free-living amoebae, *Acanthamoeba* adopts a bi-phasic life cycle, comprising a vegetative trophozoite stage and a dormant cyst stage, which at least in part might explain the wide environmental range of these protozoa (Chávez-Munguía et al., 2005). *L. pneumophila* utilizes the trophozoites for replication and the double-walled cysts as shelter to escape harsh environmental conditions (Fields, 1996).

*Acanthamoeba* replicates by binary fission and, under culturing conditions, has a doubling time reported to range from 8 to 18 h (Berk and Garduño, 2013). The amoebae are highly motile and follow a random walk pattern with a movement speed of 0.1–0.2 μm/s. In the presence of chemical signals produced by bacteria, *Acanthamoeba* will use chemotaxis to “hunt,” actively moving toward the source of these signals (Preston and King, 1984; Schuster and Levandowsky, 1996). Moreover, *Acanthamoeba* spp. are characterized by contractile vacuoles, which expel water for osmotic regulation (Bowers and Korn, 1973), and they contain glycogen storage vacuoles, lysosomes and digestive vacuoles, wherein *L. pneumophila* seems to replicate (Ulsamer et al., 1971; Bowers and Korn, 1973; Eisenreich and Heuner, 2016). Actin microfilaments adjacent to the plasma membrane of *A. castellanii* are responsible for forming protrusions (Pollard et al., 1970), and trophozoites produce short, spine-like projections at the edge of the cell, known as acanthapodia. As the amoebae are very motile, these projections are short-lived but constantly replaced, normally collapsing after less than a minute during active movement (Preston and King, 1984). *A. castellanii* also employs a diverse repertoire of putative pattern recognition receptors (PRRs), many of which with postulated orthologous functions in the innate immune systems of higher organisms (Clarke et al., 2013).

The genome of *A. castellanii* is polyploid and harbors ~15,500 compact intron-rich genes, a number of which are predicted to have been acquired through inter-kingdom lateral gene transfer (Clarke et al., 2013). The genomic complexity of *A. castellanii* remains one of the barriers to its broad utilization as a model organism. Transfection has been predominantly unsuccessful, with only one report of success (Peng et al., 2005). Furthermore, the polyploid genome organization prevents simple knockout of genes of interest, although approaches using RNA interference to knock down gene expression have been successfully used (Lorenzo-Morales et al., 2005). Overall, however, the known sequence of the *A. castellanii* genome and the increasing number of available molecular tools will strongly augment ecological and cellular research with this authentic host of *L. pneumophila*.

## *Dictyostelium discoideum*: a versatile cellular model for *Legionella* infection

In contrast to *Acanthamoeba* spp., *Dictyostelium discoideum* is not a prevalent natural host for *L. pneumophila* in the environment. This social amoeba, which naturally lives in forest soil and has a narrow temperature tolerance of 20–25°C, undergoes a complex developmental program from a single cell to become a multicellular organism (Taylor et al., 2009; Bretschneider et al., 2016). *D. discoideum* replicates by mitotic division of single amoebae. These amoeba cells live of bacteria, which are taken up by phagocytosis. Laboratory strains, e.g., Ax2, can also thrive in liquid medium through macropinocytosis. Exhaustion of the nutrient supply triggers an intricate multicellular cooperativity. In response to cAMP signals, solitary cells aggregate by chemotaxis, and ~100,000 cells form a migrating slug, which responds thermo-tactically and photo-tactically. At the final stage of morphogenesis a fruiting body is formed, consisting of a basal disc, a stalk, and spores. Accordingly, the genome of *D. discoideum* encodes morphogenetic traits such as cell-type determination, spatial patterning, cell death, and other features that are essential in multicellular but not unicellular organisms (Steinert and Heuner, 2005; Steinert, 2011; Schilde et al., 2016). The genome of *D. discoideum* is about 34 Mb and comprises six chromosomes, which range from 3.5 to 8.6 Mb in size. Most genes contain introns with conserved splice junctions. About 100 times smaller than the human genome, the *D. discoideum* genome encodes ~12,500 predicted proteins, including a large number of mammalian orthologs (Eichinger et al., 2005). In addition, *D. discoideum* is increasingly used as a model for the study of genes that in mutant form cause disease in humans (Müller-Taubenberger et al., 2013).

The completed genome sequence of *D. discoideum*, a remarkable repertoire of molecular genetic tools, and the intrinsic biological features of the amoebae allow to study many fundamental cellular processes (Bergmann and Steinert, 2015; Bretschneider et al., 2016; Hochstrasser and Hilbi, 2017). In the haploid amoeba *D. discoideum* non-essential genes can be easily disrupted or replaced by homologous recombination. Further genetic manipulation strategies include random insertion mutagenesis (restriction-enzyme-mediated integration, REMI), multiple gene deletions, Cre/LoxP-mediated recombination, RNA interference techniques, and ectopic expression of endogenous or foreign genes (Kuspa and Loomis, 1992; Chen et al., 1994; Faix et al., 2004; Kuhlmann et al., 2006; Al-Quadan and Kwaik, 2011). The large mutant collection of the *Dictyostelium* stock center (http://www.dictybase.org) (Gaudet et al., 2011), easy cultivation, amenability to diverse biochemical and biological approaches, such as *in vivo* expression of fluorescence-tagged proteins, also contributed to the present strength and versatility of the *D. discoideum* model. Recent highlights resulting from using this model host of *L. pneumophila* will be discussed below.

## Intracellular replication and competition of *L. pneumophila* in amoebae

**Figure 2:**
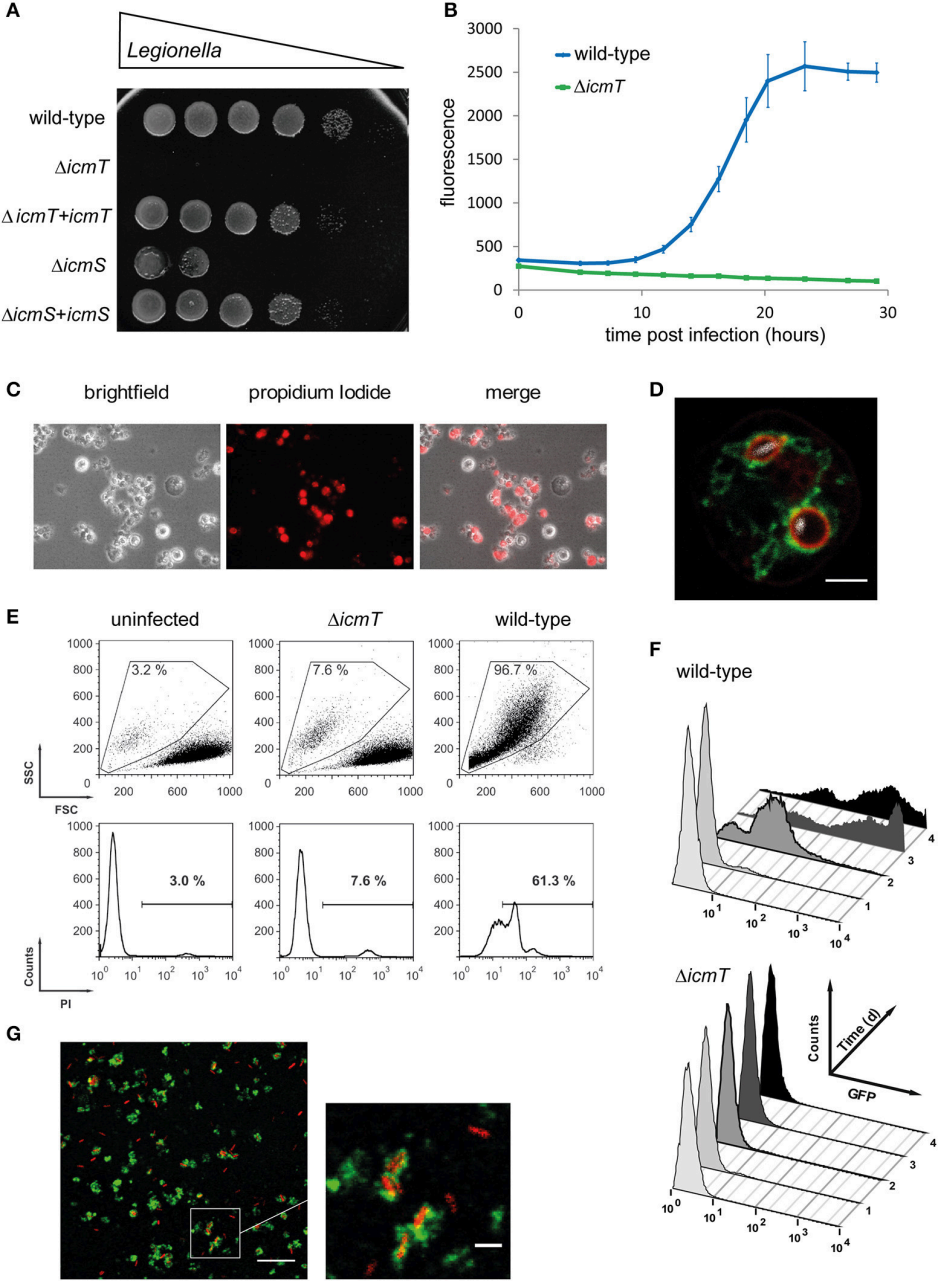
Experimental approaches to study *Legionella* infection of amoebae. **(A)** Amoeba plate test showing serial dilutions of bacterial cultures spotted on a lawn of *A. castellanii*. Virulent *L. pneumophila* is amoeba-resistant and grows in high dilutions, while mutant strains lacking a functional Icm/Dot T4SS do not (Δ*icmT*) or barely (Δ*icmS*) grow. Growth defect of the mutants is complemented by plasmid-borne expression of the corresponding genes (Δ*icmT*+*icmT*, Δ*icmS*+*icmS*). **(B)** Intracellular replication quantified by fluorescence from GFP-producing *L. pneumophila*. Virulent bacteria show an increase in fluorescence over time, while Δ*icmT* mutant bacteria do not grow. **(C)** Fluorescence microscopy images of *L. pneumophila*-infected, dying amoebae that round up, losing the characteristic spiky morphology, and take up the fluorescent dye propidium iodide. **(D)** Live-cell confocal fluorescence microscopy of *D. discoideum* strain Ax3 producing P4C-mCherry (red) and GFP-Sey1 (green), infected (MOI 10, 2 h) with mCerulean-producing *L. pneumophila* JR32 (white). Scale bar: 2 μm. **(E)** Flow cytometry of *A. castellanii* infected with *L. pneumophila* wild-type or Δ*icmT* and stained with propidium iodide. Dye uptake and changes in morphology are quantified by fluorescence and light scattering, respectively. **(F)** Flow cytometry gating on infected *A. castellanii* reveals replication of GFP-producing intracellular *L. pneumophila* wild-type but not Δ*icmT* mutant bacteria. **(G)** LCV isolation by immuno-magnetic separation and density gradient centrifugation from lysates of *D. discoideum* Ax3, infected (MOI 10, 2 h) with DsRed-producing *L. pneumophila* JR32 (red). The preparation was immuno-stained with an anti-calnexin antibody, followed by a FITC-coupled secondary antibody (green). Scale bar: 10 μm, zoom: 2 μm. Image **(A)** was reproduced with permission from Albers et al. (2005) and images **(C,E,F)** from Tiaden et al. (2007).

The amoeba plate test is a relatively simple assay to determine the ability of *L. pneumophila* mutants to form colonies in the presence of *A. castellanii* (Albers et al., 2005) (Figure 2A). A suspension of *A. castellanii* or media alone is spread onto charcoal yeast extract (CYE) agar plates, and allowed to dry. Serial dilutions of *L. pneumophila* are spotted onto the plates and incubated at 30 or 37°C for 3 days. This method allows comparing the growth of multiple strains on a single plate, in both the presence and absence of amoebae. In this manner, *L. pneumophila* mutants defective for amoebae resistance and intracellular replication can be determined in a semi-quantitative manner, alongside bacterial strains capable of suppressing these phenotypes (Albers et al., 2005). A variant of this method has been employed to screen clonal libraries of >20,000 *L. pneumophila* mutants for virulence defects (Aurass et al., 2009). In this approach, mutagenized bacteria are used to infect *A. castellanii* prior to plating.

While the amoeba plate test allows screening of strong replication phenotypes, the resulting output is not easily quantified. A more detailed analysis can be performed by measuring the number of bacteria present within or released by amoebae at given time points by determining bacterial colony forming units (CFU). *A. castellanii* or *D. discoideum* is infected with *L. pneumophila* in Ac medium or MB medium, respectively. In these media *L. pneumophila* is unable to replicate, which allows detection of intracellular replication exclusively. The host cells are then lysed by shear forces or mild detergent treatment, followed by plating on CYE agar to determine CFU. This assay has been used to assess the infection and replication of *L. pneumophila, L. dumoffii*, and *L. feelii* in *A. castellanii* (Moffat and Tompkins, 1992). Upon uptake of *L. pneumophila*, bacterial CFU drop significantly in the first few hours. Thereafter, the pathogen replicates intracellularly, leading to a 100–1,000 fold increase in bacterial numbers, and at the end of the cycle, the bacteria escape and disseminate. Bacterial amoebae resistance and intracellular replication can also be applied to screening of water samples. In this approach, samples negative for *Legionella* by normal culture techniques are incubated with amoebae, allowing intracellular growth of the bacteria to easily detectable numbers (Sanden et al., 1992).

Faster and more sensitive methods to determine intracellular replication of *L. pneumophila* in amoebae utilize an automated microtiter plate reader. This approach allows to measure the increase in optical density at 600 nm (Coil et al., 2008) or bacteria-produced fluorescence, e.g., GFP (Harrison et al., 2013, 2015a,b) (Figure 2B). To monitor intracellular growth by fluorescence increase over time, *A. castellanii* or *D. discoideum* is infected at 30/37°C or maximum at 25°C, respectively, with either GFP- or mCherry-producing *L. pneumophila* strains. To this end, we preferentially use LoFlo medium, a commercially available, complex *D. discoideum* medium that exhibits low auto-fluorescence and does not allow extracellular growth of *L. pneumophila*. The fluorescent signal for each sample is measured by microtiter plate reader at various time points post infection (p.i.). Since the fluorescence intensity is proportional to the number of bacteria present in a sample, the quantification of fluorescence increase reflects the rate of intracellular bacterial replication. Using the CFU or fluorescence assays, a number of *L. pneumophila* regulators or effector proteins, including LqsR (Tiaden et al., 2007), RidL (Finsel et al., 2013b), LppA (Weber et al., 2014a), and LegG1 (Rothmeier et al., 2013), have been found to promote intracellular replication in amoebae.

In contrast, the deletion of some other regulators (LqsA), effectors (SidC, SidM), or unknown proteins (HdeD) did not result in an intracellular growth defect of *L. pneumophila*. This might be due to robust intracellular replication of *L. pneumophila* involving redundant effectors, or simply because a gene is not required under the conditions tested. To discover weaker phenotypes of *L. pneumophila* mutant strains, we established the more sensitive amoebae competition test (Kessler et al., 2013). In this assay, *A. castellanii* is co-infected with the *L. pneumophila* parental and deletion mutant strain at a 1:1 ratio (multiplicity of infection of 0.01 each) and grown at 37°C for 15–21 days. Every third day the supernatant and lysed amoebae are diluted 1:1000, fresh amoebae are infected, and CFU are determined on agar plates containing kanamycin (to select for presence of the resistance cassette in the mutant strains) or not. Using this approach, *L. pneumophila* mutant strains lacking individual Lqs components (Kessler et al., 2013) or single effectors (LegG1, RidL, LppA, SidC) were outcompeted by the parental strain (Finsel et al., 2013b; Rothmeier et al., 2013; Dolinsky et al., 2014; Weber et al., 2014a). Thus, in the course of successive rounds of infection the fitter strain (usually the parental strain) will come to dominate the bacterial pool.

Using these assays, in addition to *L. pneumophila* mutant strains, the effect of host proteins on intracellular bacterial replication can also be assessed. Upon pharmacological inhibition of phosphoinositide (PI) 3-kinases (PI3Ks) by wortmannin or LY294002, or deletion of the kinases in *D. discoideum, L. pneumophila* replicated more efficiently, and therefore the PI3Ks restrict intracellular growth (Weber et al., 2006). In contrast, overproduction of the atlastin (Atl) homolog Sey1 in *D. discoideum* promoted intracellular growth, while production of the catalytically inactive, dominant negative mutant protein Sey1_K154A or depletion by RNAi of Atl3 in mammalian cells inhibited intracellular replication of *L. pneumophila* (Steiner et al., 2017a).

## The transcriptional response of *D. discoideum* upon infection with *L. pneumophila*

In several infection models, including *D. discoideum* and human macrophages, it has been shown that the quantitative and qualitative modulation of diverse host cell functions is crucial for efficient intracellular replication of *L. pneumophila* (Bozzaro and Eichinger, 2011; Hochstrasser and Hilbi, 2017). Accordingly, the analysis of the transcriptional changes that occur in *D. discoideum* upon infection with *L. pneumophila* revealed important aspects of the complex host-pathogen cross-talk (Farbrother et al., 2006). The experiment was performed with cDNA-microarrays, covering approximately half of the *Dictyostelium* genome, and led to the identification of 731 differentially regulated host genes in a 48 h infection with the virulent *L. pneumophila* Philadelphia-1 strain JR32. In addition, a detailed analysis of the 24 h time point p.i. of *L. pneumophila* JR32, an avirulent *L. pneumophila* Δ*dotA* mutant, or *Legionella hackeliae* (reduced virulence), uncovered 131 differentially expressed genes common to all three strains compared with uninfected amoebae. The corresponding gene products are either involved in host-specific defense mechanisms or required for successful infection and proliferation of *L. pneumophila* (Farbrother et al., 2006).

Functional annotation of the differentially regulated genes of the 48 h and 24 h time points revealed that in addition to triggering a stress response, *L. pneumophila* not only interferes with intracellular vesicle trafficking, but also subverts and exploits the host metabolism. A considerable enrichment of differentially regulated genes involved in translation, proteolysis, nucleotide metabolism, and lipid modification was observed. Moreover, *L. pneumophila* induces the expression of multiple amino-acyl t-RNA synthetases (aaRSs). Interestingly, besides the canonical coupling of correct amino acids to t-RNA molecules, aaRSs were described to play an unexpected role in splicing, apoptosis, and regulation of transcription and translation (Hausmann and Ibba, 2008; Yannay-Cohen et al., 2009). Thus, the upregulation of *D. discoideum* aaRSs upon *L. pneumophila* infection may serve a similar function in different signaling pathways. The transcriptome results also showed that infection with *L. pneumophila* results in differential expression of host genes that are involved in bacterial degradation and autophagy. Additionally, genes encoding cytoskeleton proteins were enriched ~7-fold during the early stage of infection. These findings likely reflect the uptake of bacteria and transport of the nascent phagosome inside the host cell (Farbrother et al., 2006).

In a different study it was found that infection of *D. discoideum* by *L. pneumophila* caused a decrease in mitochondrial messenger RNAs and a cleavage of the mitochondrial large subunit ribosomal RNA (LSU rRNA). The specific cleavage of the LSU rRNA in *D. discoideum* required functional *L. pneumophila* type II and type IV secretion systems. LSU rRNA cleavage was, however, not observed upon infection of *A. castellanii* or human U937 macrophages, suggesting that *L. pneumophila* might use distinct mechanisms to interrupt the mitochondrial metabolism in different hosts (Zhang and Kuspa, 2009).

In summary, the transcriptional changes in *L. pneumophila*-infected *D. discoideum* suggest complex and still poorly understood regulatory interactions between pathogen and host. In the course of establishing its replicative niche, *L. pneumophila* not only interferes with signaling processes, mitochondrial function, and intracellular vesicle trafficking, but also profoundly influences the metabolism of its host.

## Analysis of *Legionella* infection in defined *D. discoideum* mutant strains

Investigations of *D. discoideum* mutant strains have significantly contributed to our understanding of *Legionella* infection. Host cell factors implicated in host-pathogen interactions are either involved in the cellular defense or exploited by the pathogen in the course of infection. Consequently, the inactivation of distinct host factors may either favor or impede *Legionella* infection. To identify host genes crucial for *Legionella* infection, candidate gene approaches as well as untargeted mutational screens have been carried out in *D. discoideum* (Bozzaro et al., 2008; Li et al., 2009). In the candidate gene approach, either available *D. discoideum* mutants are used, which in many cases can be ordered from the *Dictyostelium* stock center (Fey et al., 2013), or novel genes of interest are disrupted, silenced, or the corresponding proteins overproduced as fluorescently tagged fusions (Bozzaro et al., 2008; Steinert, 2011; Steiner et al., 2017b). In order to dissect the complex cross-talk between *D. discoideum* and *L. pneumophila*, classical infection assays were performed, and a versatile methodology has been developed based on fluorescently tagged marker proteins, heterologously produced bacterial effectors and different visualization techniques (Eichinger and Rivero, 2006; Steinert, 2011; Steiner et al., 2017b). As select examples, we will discuss the role of the host cytoskeleton, autophagy machinery, and phospholipid metabolism in the infection process.

Most *D. discoideum* knock-out mutants of cytoskeletal proteins caused a decrease in the uptake and in the proliferation of *L. pneumophila*, indicating that an intact cytoskeleton is important (Bozzaro et al., 2013). Exceptions to this rule were the coronin A and B genes. Coronins comprise a large family of proteins that function mainly in actin cytoskeleton-associated processes. The proteins harbor a WD (Trp-Asp)-repeat domain containing seven repeats that form a seven-bladed β-propeller, adjacent to a unique region, and a C-terminal coiled coil region which mediates trimerization (Shina and Noegel, 2008). We studied uptake and replication of *L. pneumophila* in *D. discoideum* mutants where *corA* (encoding the conventional coronin), *corB* (encoding coronin 7 (CRN7)), or *vilA* (encoding villidin a member of the coronin 4 family) were disrupted. Absence of conventional coronin reduced the uptake of *L. pneumophila* and enhanced intracellular growth. Villidin-deficient cells showed an even higher reduction in uptake, and intracellular growth was strongly reduced (Fajardo et al., 2004). In contrast, uptake of *L. pneumophila* was enhanced in CRN7-deficient cells but intracellular replication was not affected. Overexpression of CRN7 on the other hand caused reduced internalization and an increased replication of *L. pneumophila*, suggesting that CRN7 negatively regulates the internalization of the bacteria. A double mutant wherein the *corA* and *corB* genes were inactivated behaved like the *corA* deletion strain (Shina et al., 2010, 2011) (Table 1). CRN7 harbors a Cdc42- and Rac-interactive binding (CRIB) domain in each of its two β-propeller domains with a preference for GDP-loaded Rac. Thus, CRN7 might keep Rac GTPases in their GDP-bound form and locally prevent the activation of the downstream targets SCAR and WASP. Loss of CRN7 hyper-activates WASP, which promotes F-actin assembly leading to increased phagocytosis (Swaminathan et al., 2015).

**Table 1:** Selected *D. discoideum* mutants implicated in *L. pneumophila* interactions.

**Host cell factor**	**Manipulation**	**Effects on infection:**		**References**
		**Uptake**	**Growth**	
ATG 9	Knockout	Down	Up	Tung et al., 2010
ATG 16	Knockout	Down	n.t.	Xiong et al., 2015
ATG 9/ATG 16	Double knockout	Down	n.t.	Xiong et al., 2015
Coronin	Knockout	Down	Up	Solomon et al., 2000; Fajardo et al., 2004
		n.t.	Up	
Coronin 7	Knockout	Up	Normal	Shina et al., 2010
	Overexpression	Down	Normal	
Coronin/coronin 7	Double knockout	Down	Up	Shina et al., 2011
Dd5P4 (OCRL)	Knockout	Down	Up	Weber et al., 2009a
PLC	Inhibitors	Down	Normal	Peracino et al., 2010
PI3K 1/2	Double knockout	Normal	Up	Weber et al., 2006
PI3K 1/2/3/4/5/6	Sextuple knockout	Down	Up	Peracino et al., 2010
PI3K 1/2/3/4/5/6/PTEN	Septuple knockout	Down	Up	Peracino et al., 2010
PTEN	Knockout	Down	Normal	Peracino et al., 2010
RpkA	Knockout	Normal	Up	Riyahi et al., 2011
Sey1	Overexpression (WT)	n.t.	Up	Steiner et al., 2017a
	Overexpression (DN)	n.t.	Down	
TBC1D5	Knockout	n.t.	Down	Bärlocher et al., 2017a
Villidin	Knockout	Down	Down	Fajardo et al., 2004

*L. pneumophila uptake and intracellular growth in D. discoideum mutants was assessed by different methods. Effects (“up” or “down”) on bacterial uptake or intracellular growth are indicated relative to the parental amoeba strain or untreated amoebae, respectively. ATG, autophagy; PLC, phospholipase C; PI3K, phosphoinositide 3-kinase class I; PTEN, phosphatase and tensin homolog; Dd5P4, D. discoideum inositol 5-phosphatase 4; OCRL, oculocerebrorenal syndrome of Lowe; RpkA, receptor phosphatidylinositol kinase A; DN, dominant negative; WT, wild-type; n.t.: not tested*.

In recent years, autophagy has been identified as an ancient and important cellular defense mechanism that targets intracellular pathogens for destruction (Sherwood and Roy, 2016). A number of pathogens are able to subvert this cellular defense, and also *L. pneumophila* interferes with the autophagy process of the host. In early studies with *D. discoideum* mutants deficient for either one of five different core autophagy genes autophagy appeared to be dispensable for intracellular replication of *L. pneumophila* at later stages of infection (Otto et al., 2004). However, infection assays with *atg9, atg16*, and *atg9/16* deletion mutants revealed a reduced uptake of *L. pneumophila* in these strains in comparison to Ax2 wild-type cells (Tung et al., 2010; Xiong et al., 2015). For the *atg9* deletion mutant it was further shown that those bacteria that entered the host were initially cleared less efficiently and at later stages of the infection multiplied more efficiently compared to the parental amoebae (Tung et al., 2010) (Table 1). It must be kept in mind that apart from its role in autophagy ATG9 likely has additional roles in trafficking, as it is localized to a number of cytoplasmic vesicular structures (Noda, 2017). Noteworthy, *atg9* and *atg16* were oppositely regulated in parental *D. discoideum* in response to infection (Farbrother et al., 2006; Tung et al., 2010).

The *L. pneumophila* Icm/Dot substrate RavZ is a cysteine protease that irreversibly cleaves lipid-conjugated Atg8 proteins on the membranes of nascent autophagic structures (Choy et al., 2012). Furthermore, the *L. pneumophila* sphingosine-1 phosphate lyase (LpSpl) targets host sphingolipid metabolism and inhibits autophagy in mouse macrophages (Rolando et al., 2016). These findings suggest that both, the host cell and the pathogen, modulate autophagy for their own benefit.

Another cellular process that is part of the defense reaction of the host and exploited by *L. pneumophila* is the PI lipid metabolism (Weber et al., 2009b; Haneburger and Hilbi, 2013). PI lipids are important for vesicle trafficking and organelle identity, since the cellular compartments are defined in part by their PI composition. The lipid head group can be phosphorylated or dephosphorylated at the 3′, 4′, and/or 5′ positions by specific PI kinases and PI phosphatases, resulting in an array of seven specific PIs (Di Paolo and De Camilli, 2006). Deletion or pharmacological inhibition of PI3Ks class I promotes intracellular replication of *L. pneumophila* and impairs the transition from tight to spacious LCVs (Weber et al., 2006). Disruption of *D. discoideum* PTEN (phosphatase and tensin homolog), a PI phosphatase antagonizing PI3Ks, reduces the uptake but does not affect proliferation of *L. pneumophila* (Peracino et al., 2010). *D. discoideum* PLC (phospholipase C) is involved in the PI metabolism through hydrolysis of PI(4,5)*P*_2_ to diacylglycerol (DAG) and inositol 1,4,5-triphosphate (IP_3_). Its inhibition dramatically reduced the engulfment of *L. pneumophila*, but had no effect on bacterial replication (Peracino et al., 2010).

Furthermore, in *D. discoideum* cells lacking the PI 5-phosphatase Dd5P4, a homolog of human OCRL (oculocerebrorenal syndrome of Lowe), bacterial replication and LCV formation occurred more efficiently. Dd5P4 localizes to LCVs via its N-terminus and might interact with the *L. pneumophila* PI(3)*P*-binding virulence factor LpnE (Weber et al., 2009a). The PI 5-phosphatase is catalytically active on the LCV, thereby increasing the PI(4)*P* available for binding of the *L. pneumophila* effector proteins SidC or SidM. An additional host protein, which plays a role in the PI metabolism is RpkA, an unusual seven-helix trans-membrane receptor with a predicted intracellular PI(4)*P* 5-kinase activity. RpkA is specific for lower eukaryotes and is recruited to nascent LCVs in *D. discoideum*. It disturbs the PI balance and thereby plays a role in the defense against *L. pneumophila*. RpkA interacts with the V-ATPase complex, but whether it can actively recruit the V-ATPase to the LCV is not known (Riyahi et al., 2011) (Table 1).

These examples illustrate the power of the *D. discoideum* system for the investigation of the host side during infection. The biological properties of this amoeba in combination with the straight-forward generation and analysis of mutant strains will continue to provide a potent and versatile model for the dissection of the complex cross-talk with *L. pneumophila*.

## Fluorescence microscopy of *Legionella*-infected labeled *D. discoideum*

The difficulty of performing genetic manipulations in *A. castellanii* implies that modifications of host cell factors, for example the production of GFP-labeled proteins, are not feasible. One of the few fluorescence assays that can be performed is the determination of *Legionella*-triggered cytotoxicity. To this end, the number of infected *A. castellanii* cells permeable to fluorescent dyes such as propidium iodide is counted, as has been done to assess the cytotoxicity of *L. pneumophila* deletion mutants (Albers et al., 2007) (Figure 2C). Given these constraints, genetically tractable amoebae such as *D. discoideum*, are more useful for cell biology and pathogen-host interaction studies, which examine the localization of specific markers to the LCV.

A plethora of genetic tools is available to study host-pathogen interactions using *D. discoideum*. In addition to DNA microarrays and targeted deletions or random mutations of genes, an extensive library of plasmids is available allowing constitutive or inducible production of fluorescently labeled probes in *D. discoideum* (http://wiki.dictybase.org/dictywiki/index.php/Vectors). Specifically, various expression vectors for N- or C-terminal fusions with green- or red-fluorescent proteins are available. These include extrachromosomal (Levi et al., 2000), extrachromosomal inducible expression (Veltman and van Haastert, 2013), or integrating plasmids (Manstein et al., 1995), as well as vectors for efficient generation of gene knockouts using one-step cloning (Wiegand et al., 2011). For the production of fluorescent probes, *D. discoideum* is transformed with one or multiple plasmids by electroporation (Weber et al., 2014b). Transformants are isolated by antibiotic selection and form microcolonies within 7–12 days after transformation.

A GFP-fusion protein commonly used in *D. discoideum* is calnexin-GFP, an ER-specific type I transmembrane protein and well-established LCV marker (Fajardo et al., 2004; Weber et al., 2006; Ragaz et al., 2008; Dolinsky et al., 2014). Within 2 h after uptake, calnexin accumulates on the membrane of LCVs containing virulent *L. pneumophila*, but not avirulent Δ*icmT* mutant bacteria, and to a significantly smaller extent on vacuoles harboring Δ*sidC* mutants (Ragaz et al., 2008; Dolinsky et al., 2014; Weber et al., 2014b). Using *D. discoideum* cells producing fluorescently labeled proteins, numerous other host proteins were found to localize to the LCV. These include small GTPases of the Rab family (Urwyler et al., 2009; Hoffmann et al., 2014), Rap1 (Schmölders et al., 2017), and Ran (Rothmeier et al., 2013), the ER tubule-resident large GTPase Atl3/Sey1 (Steiner et al., 2017a) (Figure 2D), as well as components implicated in the retrograde vesicle trafficking pathway, such as the *D. discoideum* OCRL homolog Dd5P4 (Weber et al., 2009a) and subunits of the retromer complex (Finsel et al., 2013a).

A caveat of microscopy methods using fixed samples is that the fixation techniques might alter the morphology of cellular structures such as membranes, vesicles, or microtubules. Time-lapse imaging of living cells on the other hand is optimal to follow cellular dynamics over time. A well-studied process is the PI conversion on the LCV membrane during infection (Weber and Hilbi, 2014; Weber et al., 2014b). *L. pneumophila*-infected *D. discoideum* producing various PI probes, including PH_CRAC_-GFP for PI(3,4,5)*P*_3_ and PI(3,4)*P*_2_, 2 × FYVE-GFP for PI(3)*P* or GFP-P4C_SidC_ for PI(4)*P*, were imaged live for time-lapse analysis (Weber et al., 2014b). Immediately upon bacterial uptake, the phagosome containing *L. pneumophila* transiently accumulates PI(3,4,5)*P*_3_ for up to 1 min, and within that time-span, PI(3)*P* is acquired in a Icm/Dot independent manner. During the following 2 h, LCVs containing virulent *L. pneumophila* gradually replace PI(3)*P* with PI(4)*P*, whereas Δ*icmT* vacuoles remain PI(3)*P* positive (Weber et al., 2014b). Using dually labeled *D. discoideum*, the P4C-mRFPmars signal was spatially separated from calnexin-GFP-positive membranes and maintained for at least 8 h. Manipulation of the LCV PI pattern is of great importance for the bacterium. The PI 5-phosphatase Dd5P4 as well as PI3Ks and PI4KIIIβ have been implicated in this process (Brombacher et al., 2009; Weber et al., 2009a,b).

Live-cell imaging was also employed to assess the LCV and microtubule dynamics in infected *D. discoideum* producing calnexin-GFP (Rothmeier et al., 2013) or GFP-α-tubulin (Simon et al., 2014). The *D. discoideum* strains were infected with DsRed-producing *L. pneumophila*, wild-type, Δ*legG1*, or the complemented mutant. LegG1 is an Icm/Dot-translocated RCC1-like-repeat effector protein, which activates the small GTPase Ran and increases cellular RanGTP levels (Rothmeier et al., 2013). Ran is a member of the Ras superfamily of small GTPases and controls various cellular processes, including nucleo-cytoplasmic transport (Stewart, 2007), assembly of the mitotic spindle apparatus (Goodman and Zheng, 2006; Clarke and Zhang, 2008), and the dynamics of non-centrosomal microtubules (Yudin and Fainzilber, 2009). LCV motility as well as microtubule stability was significantly reduced in *D. discoideum* infected with Δ*legG1* mutant bacteria.

## Quantitative assessment of *Legionella* infection by (imaging) flow cytometry

Flow cytometry is a high-throughput technique that allows a simultaneous multi-parameter analysis of single cells. Large data sets, high resolution, accuracy, reproducibility, and sensitivity are major advantages of this approach to study uptake, cytotoxicity, and intracellular replication of *L. pneumophila* (Tiaden et al., 2013). The physiological status and integrity of a cell is reflected in the forward scatter/sideward scatter pattern (corresponding to cell size and “granularity,” respectively), and membrane integrity can be tested by staining cellular DNA with the fluorescent dye propidium iodide, which is recorded as “cell-associated fluorescence.”

Flow cytometry can be used to study in a quantitative manner *Acanthamoeba* phagocytosis. To this end, the rate and degree of uptake of fluorescent beads are assessed, i.e., the ratio of fluorescent vs. non-fluorescent amoebae (Avery et al., 1995), or, analogously, the infection by fluorescently labeled *L. pneumophila* is determined (Tiaden et al., 2013). Uptake of *L. pneumophila* is scored immediately after infection by determining an “uptake index,” which is calculated from the signal-strength and percentage of amoebae infected with GFP-producing bacteria (Harf et al., 1997; Tiaden et al., 2013). Hence, cytotoxicity of *L. pneumophila* can be assessed as a change in size and shape of *A. castellanii*, measured by forward/sideward light scattering, as well as by loss of membrane integrity, as determined by the increase in permeability to propidium iodide (Tiaden et al., 2007, 2008) (Figure 2E).

Moreover, using flow cytometry and GFP-producing bacteria, not only the uptake efficiency can be determined, but also intracellular replication in *A. castellanii* or *D. discoideum* can be followed over several days. This approach has been utilized to examine the uptake and replication defects of *L. pneumophila* deletion mutants lacking components of the *Legionella* quorum sensing (Lqs) system: LqsR (Tiaden et al., 2007), LqsA or LqsS (Tiaden et al., 2010), LqsT or LqsS-LqsT (Kessler et al., 2013), as well as the entire *lqs* gene cluster (Tiaden et al., 2008) (Figure 2F). Analogously, flow cytometry can be employed to assess uptake and replication phenotypes of *D. discoideum* deletion mutant strains lacking PI3Ks (Weber et al., 2006) or Dd5P4 (Weber et al., 2009a). Finally, in addition to its use as a research tool, flow cytometry has also been developed as a diagnostic method to rapidly screen for *Legionella* contamination of bathing facilities and other water sources (Taguri et al., 2011).

In a technique termed imaging flow cytometry (IFC), flow cytometry is combined with fluorescence microscopy, enabling fast quantification of microscopic images. Using this approach, the high-throughput capacity and information about cell size, volume, and shape provided by the flow cytometer is combined with high-resolution spatial localization of fluorescent proteins of interest provided by a microscopic image, resulting in non-biased and quantitative, large datasets (Barteneva et al., 2012; Johansson et al., 2015). By means of specialized software, the acquired cellular images can be analyzed using bi-variant plots and histograms, in parallel with a sequential gating strategy.

Recently, we employed IFC and *D. discoideum* ectopically producing GFP- and mCherry-labeled fusion proteins to assess the accumulation of specific host proteins on LCVs in whole cells (Schmölders et al., 2017; Steiner et al., 2017a) or homogenates (Bärlocher et al., 2017a). Thus, active small GTPase Rap1 was found to localize to a higher extent to LCVs containing the parental *L. pneumophila* strain Lp02 as compared to LCVs harboring the “pentuple” mutant strain (Schmölders et al., 2017), which lacks five gene clusters encoding 31% of the effector proteins (O'Connor et al., 2011). The method was also utilized to quantify the recruitment of calnexin-GFP or GFP-Sey1 to PI(4)*P*-positive LCVs labeled with P4C-mCherry. Dually labeled *D. discoideum* amoebae producing P4C-mCherry and GFP-Sey1 or GFP-Sey1_K154A were infected, and co-localization of PI(4)*P* with *L. pneumophila* was analyzed for 10,000 events per time point p.i. IFC confirmed and quantified the fluorescence microscopy finding that PI(4)*P* accumulates at the LCV in a Sey1-independent manner. Finally, the Rab7 GAP TBC1D5 was scored on LCVs. However, in this case lysates of infected *D. discoideum* had to be prepared to reduce background and increase sensitivity (Bärlocher et al., 2017a). To this end, *D. discoideum* amoebae producing in tandem GFP-TBC1D5 and the endosomal marker AmtA-mCherry were infected with mPlum-producing *L. pneumophila*, and the localization of TBC1D5 was analyzed by IFC in cell homogenates. For each condition, over 3,000 cells were analyzed, and TBC1D5 was found to localize to wild-type-, but not Δ*icmT*-containing LCVs.

## Chemotactic migration of *Legionella*-infected *D. discoideum*

In a process termed chemotaxis, phagocytes migrate in a directed manner toward an attractant. Thus, *D. discoideum*, macrophages or neutrophils sense and respond to cAMP, the chemokines CCL5, and tumor necrosis factor (TNF)-α, or the formyl-methionyl-leucyl-phenylalanine (fMLP) peptide, respectively. The migration of eukaryotic cells critically depends on microtubule polarization and dynamics (Etienne-Manneville, 2013), which in turn is controlled by the small GTPase Ran.

Given the impact of the Icm/Dot substrate LegG1 on Ran GTPase activation and microtubule dynamics (Rothmeier et al., 2013), we analyzed the role of the *L. pneumophila* T4SS and LegG1 on host cell motility (Simon et al., 2014). Using *D. discoideum* amoebae or mammalian immune cells in under-agarose or Boyden chamber migration assays, *L. pneumophila* was found to inhibit cell migration in an Icm/Dot-dependent manner. *D. discoideum* infected with virulent *L. pneumophila* showed a substantially reduced migration when compared to amoebae infected with Δ*icmT* mutant bacteria. The migration inhibition observed was not due to an uptake defect or cytotoxicity. Interestingly, *D. discoideum* infected with the Δ*legG1* mutant strain was hyper-inhibited for directed migration in the under-agarose assay, which was reverted upon overproduction of LegG1 to an extent comparable to amoebae infected with wild-type bacteria. Single cell tracking indicated that the directionality as well as the velocity of *D. discoideum* infected with the parental strain or Δ*legG1* is impaired. Thus, the Ran activator LegG1 promotes cell motility by stabilizing microtubules and thereby might antagonize the possibly deleterious impact of other *L. pneumophila* Icm/Dot substrates on the host cytoskeleton.

Recently, the effect of the small signaling molecule LAI-1 (*Legionella* autoinducer-1, 3-hydroxypentadecane-4-one) on chemotactic migration of *D. discoideum* was analyzed (Simon et al., 2015). This study was based on the observations that *L. pneumophila* lacking the autoinducer synthase LqsA no longer impaired the migration of infected amoebae, and a Δ*icmT* mutant strain overproducing LqsA inhibited cell migration, obviously in an Icm/Dot-independent manner. Synthetic LAI-1 dose-dependently inhibited the migration of *D. discoideum* in the micromolar range. RNA interference with epithelial cells subjected to a scratch (“wound closure”) assay revealed that LAI-1 signaling requires the scaffold protein IQGAP1, the small GTPase Cdc42 as well as the Cdc42-specific guanine nucleotide exchange factor ARHGEF9, but not other modulators of Cdc42, or RhoA, Rac1 or Ran GTPase. Thus, the *L. pneumophila* signaling molecule LAI-1 produced either in the LCV or added exogenously promotes inter-kingdom signaling.

In summary, *L. pneumophila* modulates the motility of eukaryotic cells not only by means of Icm/Dot substrates, but also through the Lqs system and the small signaling molecule LAI-1 (Personnic et al., 2017). At present, it is unclear what the benefit of host cell migration inhibition for *L. pneumophila* might be. The phenomenon might simply be an indirect effect of perturbation of trafficking pathways and cytoskeletal dynamics. More intriguingly, however, it might also reflect the fact that macropinocytosis competes with migration of *D. discoideum*, two processes balanced by PI(3,4,5)*P*_3_ (Veltman, 2015). Hence, by inhibiting cell migration, *L. pneumophila* might promote its macropinocytic uptake by the amoebae.

## Cell biological, biochemical, and proteomics analysis of purified intact LCVs

Intact LCVs from *D. discoideum* can be purified by a straight-forward two-step protocol involving immuno-affinity enrichment using an antibody against the Icm/Dot substrate SidC, which selectively decorates the pathogen vacuole, and a secondary antibody coupled to magnetic beads. This first step is followed by conventional Histodenz gradient density centrifugation, yielding intact LCVs in high purity and yield (Urwyler et al., 2010; Hoffmann et al., 2012, 2013) (Figure 2G). Using *D. discoideum* producing the LCV marker calnexin-GFP, infected with red fluorescent *L. pneumophila*, the LCV purification procedure can be easily followed by fluorescence microscopy.

Proteomics analysis of these purified LCVs revealed the presence of 560–1150 host proteins (Urwyler et al., 2009; Hoffmann et al., 2014; Schmölders et al., 2017). Amoebae proteins identified include small Rab GTPases, Rap1, Ran, and its effector Ran binding protein 1 (RanBP1). Moreover, large GTPases, components of the endosomal and late secretory trafficking pathways, as well as protein or lipid kinases and phosphatases were identified, many of which had not been implicated in LCV formation before. The accumulation of several of these host proteins on the LCV was confirmed by fluorescence microscopy using *D. discoideum* strains producing the corresponding GFP-fusion proteins (Urwyler et al., 2009; Finsel et al., 2013a; Rothmeier et al., 2013; Hoffmann et al., 2014; Schmölders et al., 2017; Steiner et al., 2017a). While some of these host factors accumulate on LCVs in an Icm/Dot-dependent manner (e.g., Rab1,−7,−8,−14, Rap1, Ran, RanBP1, Sey1), others localize to the pathogen vacuole Icm/Dot-independently (e.g., retromer subunits).

Preparations of intact LCVs can also be utilized for biochemical experiments (Steiner et al., 2017a). To this end, *D. discoideum* producing calnexin-GFP, GFP-Sey1, or catalytically inactive GFP-Sey1_K154A were infected with mCherry-producing virulent *L. pneumophila*. LCVs were purified according to the two-step protocol described above, and subsequently, the effect of GTP, GDP or the non-hydrolysable GTP analog Gpp(NH)p was analyzed by fluorescence microscopy. Interestingly, the purified LCVs aggregated and expanded their size about 2-fold in a Sey1- and GTP-dependent manner. This experiment revealed in a reductionist system the importance of Sey1 activity for LCV aggregation and circumferential ER remodeling.

## Screen for antivirulence compounds in *Legionella*-infected amoebae

Antibiotic resistance of human pathogens is an ever-rising and global problem. The approach to develop new bactericidal or bacteriostatic antibiotics is compromised by a high selection pressure on pathogens to become resistant against these compounds. A more efficient method is to target the virulence and the ability of bacteria to infect the host, rather than targeting directly the capacity to multiply. Such an “anti-virulence” strategy aims at specific mechanisms that promote bacterial pathogenicity, including metabolic pathways, binding to and uptake by host cells, signaling, and vesicle trafficking pathways, or the secretion and mode of action of toxins. There is the hope that anti-virulence compounds reduce the evolutionary pressure to develop drug resistance, and by selectively targeting virulent bacteria, spare the normal host microbiota.

Another major obstacle in developing novel antibiotic or anti-virulence compounds is the limited compound accessibility of intracellular bacteria, reflected in compromised bioavailability of a compound. To efficiently address these problems, and given the similarities between amoebae and human immune phagocytes such as macrophages or neutrophils (Hilbi et al., 2007; Escoll et al., 2013; Hoffmann et al., 2013), we established *A. castellanii* as a robust and high-throughput compatible model system to screen for anti-virulence compounds. To this end, the amoebae were seeded into 96-well plates and infected with GFP-producing *L. pneumophila*. The progress of intracellular growth correlates with an increase in fluorescence (Harrison et al., 2013, 2015b). The medium chosen minimizes auto-fluorescence and growth of the amoebae, as well as extracellular growth of the bacteria. Under these conditions, bacterial replication typically comprised a lag and a replicative phase, after which all amoebae were dead and bacterial fluorescence remained constant. Upon addition of low molecular weight compounds to infected amoebae, we identified palmostatin M—a β-lactone inhibitor of eukaryotic Ras depalmitoylases and Ras signaling (Hedberg et al., 2011; Rusch et al., 2011)—as an inhibitor of intracellular growth of *L. pneumophila* as well as of *Mycobacterium marinum* and *Mycobacterium tuberculosis* (Harrison et al., 2013; Kicka et al., 2014).

Moreover, using this *A. castellanii*—*L. pneumophila* infection assay, we screened a highly diverse, pathway-based chemical library, referred to as the Sinergia library (Harrison et al., 2015a). Structure-activity relationship (SAR) studies using variants of a hit compound thus identified revealed essential structural features, namely a triple-ring scaffold with a central triazine moiety, substituted by two piperidine or pyrrolidine rings in positions 3 and 5, and by an amine group bearing a single aliphatic chain moiety in position 1. The most effective compound, ZINC00615682, inhibited intracellular growth of *L. pneumophila* with an IC_50_ of ~20 nM in *A. castellanii* and somewhat less efficiently in *D. discoideum* or macrophages (Harrison et al., 2015a). These results validate the amoebae-based screen and demonstrate that a corresponding SAR analysis allows the identification of novel inhibitors of intracellularly growing *L. pneumophila* and other bacteria.

## Conclusions and outlook

Intense research over the last years revealed that amoebae not only represent an important ecological niche of the environmental bacterium *L. pneumophila*, but are also versatile and powerful models for the molecular and cellular analysis of host-pathogen interactions. The natural host *A. castellanii* is permissive for *L. pneumophila* over a range of different temperatures, and this robust system is useful to assess bacterial mutant phenotypes, as well as to screen for anti-virulence compounds. The social amoeba *D. discoideum* is genetically tractable and allows analysis of host mutant strains. Dually labeled *D. discoideum* strains are ideally suited for (live-cell) fluorescence microscopy, imaging flow cytometry, cell migration assays, or LCV isolation. Purified intact LCVs can be further analyzed by proteomics and cell biological or biochemical tests. Obvious limitations of the amoebae models are the apparent lack of key components of cell-autonomous innate immunity pathways, such as caspase family proteases and the transcription factor NF-kB (Eichinger et al., 2005; Clarke et al., 2013). On the other hand, amoebae models are useful for the analysis of host-pathogen interactions beyond *Legionella* infection. Accordingly, *Acanthamoeba* spp. and *D. discoideum* can serve to assess various aspects of cellular virulence for a large number of pathogenic bacteria including *Mycobacterium* spp., *Burkholderia* spp., and *Vibrio cholerae* (Hilbi et al., 2007; Cosson and Soldati, 2008). Hence, amoebae and other protozoa will continue to provide valuable insights into important aspects of the ecology and virulence of amoeba-resistant environmental bacterial pathogens.

## Author contributions

ALS, CFH, LE, MS, and HH wrote the manuscript.

### Conflict of interest statement

The authors declare that the research was conducted in the absence of any commercial or financial relationships that could be construed as a potential conflict of interest.

## Abbreviations

Icm/Dot: intracellular multiplication/defective organelle trafficking
LCV: *Legionella*-containing vacuole
OCRL: oculocerebrorenal syndrome of Lowe
PI: phosphoinositide
T4SS: type IV secretion system
TGN: *trans*-Golgi network.
